# Hepatoprotective effect of Quercetin supplementation against Acrylamide-induced DNA damage in wistar rats

**DOI:** 10.1186/s12906-016-1322-7

**Published:** 2016-08-30

**Authors:** Sabah Ansar, Nikhat Jamal Siddiqi, Seema Zargar, Majid Ahmad Ganaie, Manal Abudawood

**Affiliations:** 1 Clinical Laboratory Sciences, College of Applied Medical Science, King Saud University, Riyadh, Saudi Arabia; 2 Biochemistry Department, College of Science, King Saud University, Riyadh, Saudi Arabia; 3 Department of Pharmacology, College of Pharmacy, Prince Sattam Bin Abdulaziz University, Al-Kharj, Saudi Arabia

**Keywords:** Quercetin, Oxidative stress, Antioxidants, DNA damage, Antioxidants

## Abstract

**Background:**

Quercetin (QR), is a polyphenolic flavonoid compound which is found in large amounts in certain foods, and protects against oxidative stress. The current study was conducted to determine whether Quercetin can possibly exert hepatoprotective and antioxidant activity against acrylamide (ACR) induced toxicity in rats.

**Methods:**

Four groups of Wistar rats consisting of six rats each: (i) control group; (ii) ACR treated group (50 mg/kg bw); (iii) QR group: rats were treated with QR (10 mg/kg bw); (iv) QR (10 mg/kg bw) was given i.p. for 5 days followed by ACR (50 mg/kg bw) on 5th day (single dose).

**Results:**

ACR caused an elevation in 8-OH guanosine level and a reduction in Glutahione S-transferase (GST) activity. Administration of QR significantly protected liver tissue against hepatotoxic effect of acrylamide from amelioration of the marker enzyme (*p* < 0.05) and DNA damage (*p* < 0.01) as evident by comet assay and, besides some indices of histopathological alterations.

**Conclusion:**

It is concluded that QR could protect the liver against DNA damage induced by ACR probably is thus capable of ameliorating ACR-induced changes in the rat livers.

## Background

Several studies have reported that acrylamide (ACR) is formed in heat-treated food mainly containing car bohydrates [1–7]. ACR has been reported to form acrylamide-protein adducts in laboratory animals [3]. Earlier carcinogenic action of ACR has been reported in detail [8]. Recently, not only major metabolic pathways and enzymes of ACR have been summarized, but also the inter individual and the interspecies differences of ACR metabolism among humans, rats and mice have been reported [9].

Due to ACR exposure damage of biological macromolecules and disruption of normal metabolism leads to oxidative stress and imbalance in antioxidant activity [10]. Oxidative stress causes enhanced generation of reactive oxygen species (ROS) and depletion of antioxidant defense system in the tissues. ROS can stimulate free radical chain reactions, leading to the enhancement of lipid peroxidation [11].

Plants contain numerous polyphenols, which have been shown to reduce inflammation and thereby increase resistance to disease [12]. Flavonoids are present in high concentration, as polyphenols in vegetables, fruits, and beverages [13–15]. Flavonoids are known to have anti inflammatory [16], anti-allergic [17], cardio protective [18], and anti-cancer activities [19]. Also, flavonoids protect against DNA damage in certain oxidative stress conditions [20].

Quercetin is one of the most common flavonoids in the diet and exhibits therapeutic potential, including hepatoprotection and the inhibition of liver fibrosis, against many diseases [12, 21, 22]. It contains a number of phenolic hydroxyl groups that have strong antioxidant activity [15, 23–26]. Moreover, QR has been shown to protect against carbon tetrachloride, ethanol, and paracetamol-induced hepatotoxicity [27]. Our recent studies have shown that quercetin can restore against oxidative damage against acrylamide induced neurotoxicity [28]. Additionally, recent published reports have shown protective effect of QR against various toxic insults [29–32]. In this study, the effect of QR on acrylamide caused hepatotoxicity in rats has been investigated.

## Methods

### Chemicals

ACR, QR, and other reagents were bought from Sigma–Aldrich Chemicals Co., St. Louis, USA. Quercetin (Sigma) was resuspended immediately before administration in a 2 % tween aqueous solution. ACR was dissolved in saline and/or distilled water.

### Animals and experimental procedures

Male wistar rats weighing approximately 200–220 g were procured. Animal utilization protocols were performed in accordance with the guidelines provided by the Experimental Animal Laboratory and approved by the Animal Care and Use Committee. All the animals used in this study were placed in cages in an air conditioned room maintained at 12 h light/dark cycle.

Study design: Twenty-four rats were randomly divided into 4 groups (6 rats in each group). Group I received saline (0.85 % NaCl i.p) at 10 ml/kg bodyweight. Group II received ACR at a dose of 50 mg/kg b.w. Group III received pretreatment with QR -10 mg/kg body weight, and groups IV received the pretreatment with QR -10 mg/kg body weight. After the last treatment with QR, the rats of groups IV received a single i.p. injection of ACR at 50 mg/kg body weight. Forty-eight hours later, the rats were sacrificed. The dose of QR and ACR used in the present study was in accordance with previous study, respectively [33, 34]. The livers were excised, weighed, and divided for histological and biochemical analyses.

### Biochemical analysis

The liver homogenates were centrifuged at 3000 rpm for 10 min at 4 °C. The supernatants were used for the various biochemical determinations.

Liver homogenates were analyzed for Glutathione S-transferase (GST) measured by Biovision assay kit, DNA damage by comet assay and 8-OH deoxyguanosine (8-OHdG) by ELISA kit (Abnova, cat: 1221).

### Histological examinations

Small pieces of liver tissue were used for histopathological studies. Fixed tissues were dehydrated in serial ethanol series, trimmed, embedded in paraffin, sectioned into 2-μm sections and stained with hematoxylin and eosin (H&E). Morphological examination was conducted under a light microscope (Nikon Eclipse E600).

### DNA damage assay

The DNA damage evaluation was performed by single cell gel electrophoresis (comet) assay as described by [35]. For visualization of DNA damage, observations were made of Ethedium Bromide -stained DNA using a 40x objective on a fluorescent microscope. Generally, 50–100 randomly selected cells were analyzed per sample.

### Statistical analysis

Results were analyzed using SPSS software and values are given as arithmetic means standard error of the mean (S.E.M.). Data was statistically analyzed by using one-way analysis of variance (ANOVA) followed by Dunnett’s multiple comparison tests.

## Results

### Assessment of Glutathione S-Transferase (GST) activity

ACR exposure significantly decreased GST activity in the liver cells (~30 %), and this was prevented in the QR + ACR treated group (Table 1). QR pretreatment elevated the activity of GST significantly (*p* < 0.05) compared to the ACR group-2. GST activity was no different between QR alone and control groups.

**Table 1:** The effect of QR on 8-OHDG and Glutathione-peroxidase activity induced by ACR

Treatment (grps)	8OHDG (ng/ml)	Glutathione S-transferase (GST) (U/100 mg protein)
Control-G1	6.21 ± 0.31	34.32 ± 5.21
ACR-G2	12.28 ± 0.65*	24.47 ± 8.49*
QR-G3	6.44 ± 0.43	33.88 ± 4.12
QR+ ACR-G4	8.89 ± 0.47**	30.17 ± 5.19**

Values were expressed as mean ± SD, *n* = 6

*P < 0.05 and **P < 0.01

### Assessment of 8-OH dG levels

The levels of 8-OHdG was significantly elevated in liver tissues of the rats treated with ACR (*p* < 0.05). QR significantly decreased 8-OHdG levels as compared with ACR-treated rats (*p* < 0.05) (Table 1). There was no significant difference between the control group1 and QR group 3.

### Assessment of DNA damage

**Fig. 1:**
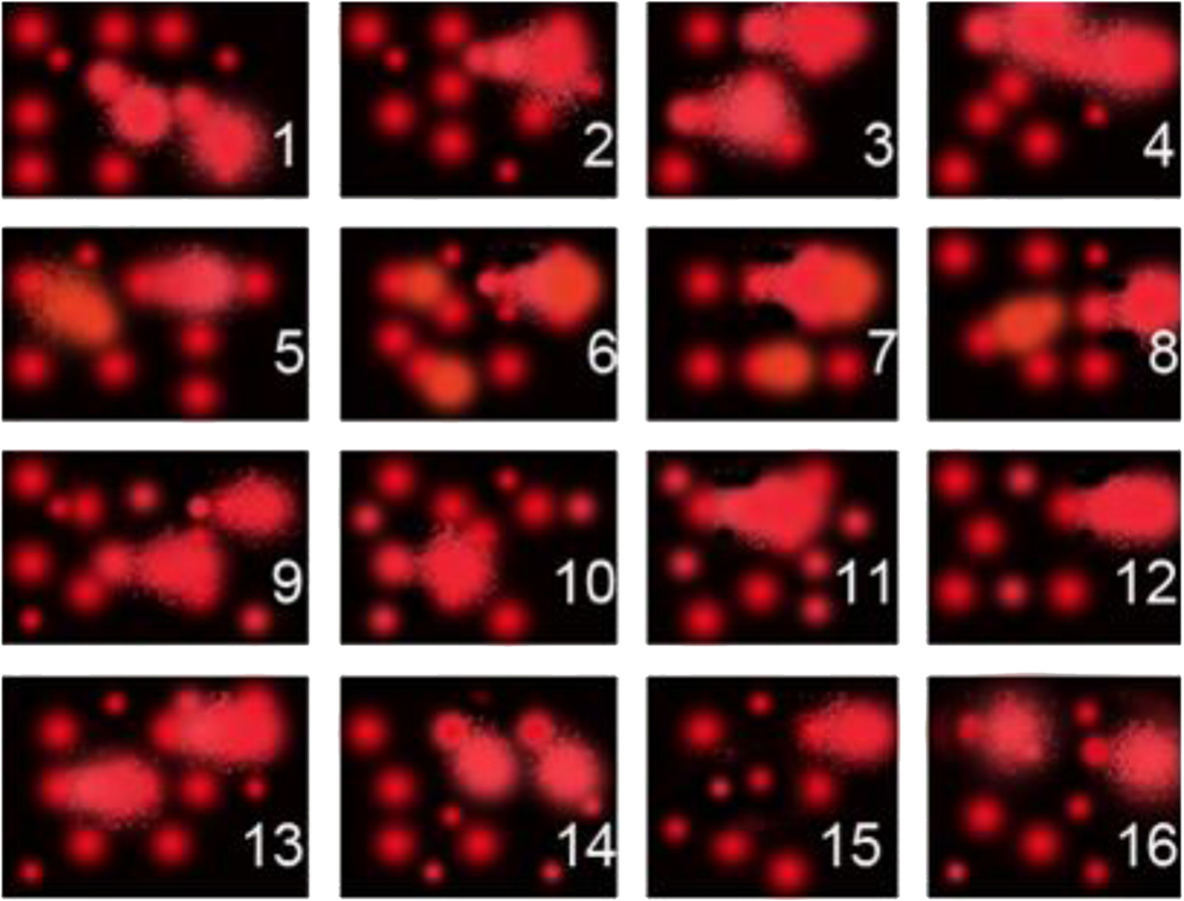
The effects of QR on DNA damage induced by ACR : 1–4 Control; 5–8 ACR; 9–12 QR; 13–16 QR+ ACR

The DNA damage was expressed as tail length, tail DNA, and tail moment in the liver Fig. 1, Table 2. DNA damage was increased significantly in the ACR group compared to the control (*p* < 0.05). QR reduced DNA damage significantly as observed in the ACR + QR group as compared to the ACR group (*p* < 0.01). There was no significant difference in DNA damage between the control group and the QR + ACR group.

**Table 2:** The effect of QR on DNA damage induced by ACR

Treatment grps	DNA Trail intensity %	DNA Tail length μm	DNA Tail moment
Control-G1	5 ± 0.41	1.58 ± 0.12	2.82 ± 0.35
ACR-G2	11.5 ± 0.23	3.37 ± 0.23*	12.43 ± 0.32*
QR-G3	6.5 ± 0.51	2.5 ± 0.35	6.22 ± 0.31
QR + ACR + G4	8 ± 0.36	2.62 ± 0.61**	5.95 ± 0.21**

Values were expressed as mean ± SEM, *n* = 6

*P < 0.05 and **P < 0.01

### Histopathological findings

**Fig. 2:**
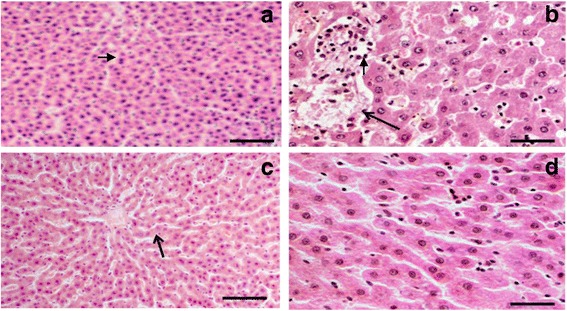
T.S. of rat livers: **a** Control: showing normal structure of hepatocytes, **b** ACR treatment showing disarrangement in cytoplasm, **c** QR treatment showing normal cellular structure (**d**) QR treatment before ACR administration showing normal hepatic cells. Scale bar: 50 μ

Figure 2 shows liver sections: Control I-A, showing normal orientation of hepatic parenchyma. Liver sections show disarrangement and mild degeneration of cells in cytoplasm from group II-B. Liver sections from group III-C and group IV-D show normal hepatic cells, and sinusoid spaces as compared to group II-B.

## Discussion

Food during baking and frying at high temperatures leads to formation of acrylamide at higher levels [36]. Recent studies have reported that a fried potato dish as large contributor to acrylamide exposure [37]. Furthermore, acrylamide has been reported as a carcinogen showing hazardous effects [38]. Acrylamide’s mechanism of action is greatly enhanced as it can be readily absorbed through the intestinal tract. Present study demonstrates that QR protects against DNA damage indicating that QR possesses DNA-protective properties. These findings are in accordance with other studies that used quercetin or other antioxidant substances, such as rutin, nacetylcysteine, and vitamins E and C, all of which decreased the severity of hepatic fibrosis [39–43].

The GST enzyme catalyzes the conjugation of the reduced form of glutathione (GSH) to xenobiotic and protect cells and tissues against oxidative stress. A reduction of GST activity was observed in homogenized liver in ACR treated group, and this reduction was blocked after quercetin administration in this study. These findings support the hypothesis that QR exerted a protective effect in vivo [29]. The histopathological profile of liver damage demonstrates that pretreatment of QR in ACR-treated group exhibits less damage to the hepatic cells as compared to the rats treated with the toxic group. It could be suggested that QR scavenges free-radical generation and inhibits ACR–induced injury in hepatic tissues.

Results in this study show that ACR induced DNA damage was significantly decreased after the treatment of QR. The antioxidant effects of QR may be due to flavonoid’s high diffusion into the membranes allowed it to scavenge oxy radicals at several sites throughout the lipid bilayer or its pentahydroxyl flavones structure allowed it to chelate metal ions via the orthodihydroxy phenolic structure, thereby scavenging lipid alkoxyl and peroxyl radicals [19, 44] or flavanoids might be also involved in the indirect induction of detoxifying genes [25, 27, 45, 46], which might promote detoxification of ACR and decrease their toxicity. Enhanced chemiluminescence studies haves shown antioxidant role of flavonoids and other polyphenols found in tea [47]. Some recent studies also support the finding that QR has also been shown to suppress toxicity and oxidative stress in vivo and in vitro [48–52].

Once ingested ACR can interact with other proteins at the cellular level and bind to DNA to form adducts [38]. It has been shown earlier that QR as flavanoides may bind DNA at sites that would normally react with the active metabolites of carcinogen [18, 20, 53]. Recently, in vivo genotoxicity assessment of acrylamide has been shown and it is reported that genotoxicity of ACR is tissue specific [54]. Acrylamide can cause gene interactions and chromosomal aberrations and has been classified as being genotoxic.

## Conclusions

In conclusion, the study revealed that ACR induces toxicity by increasing DNA damage and decreasing the glutathione S transferase activity. QR protects ACR toxicity indicating that QR possesses a spectrum of antioxidant and DNA-protective properties. However, further studies are required to elucidate the precise mechanisms of protection of QR against ACR toxicity.
